# Dengue Fever in a Neonate: A Case Report

**DOI:** 10.31729/jnma.8099

**Published:** 2023-03-31

**Authors:** Chaitanya Darshan Bhattarai, Birendra Kumar Yadav, Rabin Basnet, Manish Karki, Shanta Chauhan

**Affiliations:** 1 Department of Pediatrics, Nepal Medical College and Teaching Hospital, Jorpati, Kathmandu, Nepal; 2 Telemedicine Center COVID-19 Unified Central Hospital, Bir Hospital, Kanti Path, Kathmandu, Nepal; 3 Department of Emergency Medicine, Himal Hospital, Thirbum Marg, Kathmandu, Nepal

**Keywords:** *case report*, *dengue fever*, *neonates, Nepal*

## Abstract

Dengue is one of the most common viral infections affecting the general population in endemic areas annually. However, it is barely reported in newborns owing to a widespread belief that they are protected from severe viral infections in the first six months of life by the presence of maternal antibodies. Here we present a case of a 23-day-old male infant born to primigravida with dengue fever with the post-natal transmission of infection. He presented with complaints of fever for three days. On general examination, red-coloured pinpoint macular rashes were observed bilaterally on lower limbs. No significant findings were present on systemic examination. On routine sepsis workup, thrombocytopenia was present. Acknowledging the endemicity and expanding dengue cases, NS1 antigen and antibody IgM and IgG of the baby were tested which came positive for antigen and IgM antibody. Even so, the mother was asymptomatic with NS1 antigen, IgG and IgM antibodies negative with a normal range of platelet count.

## INTRODUCTION

Dengue is an infectious disease caused by dengue virus and is transmitted classically by bite of female *Aedes aegypti* mosquito. Dengue virus (DV) infection can have a range of presentation from dengue fever to dengue haemorrhagic fever/dengue shock syndrome (DHF/DSS) that is characterized by systemic capillary leakage, thrombocytopaenia and hypovolaemic shock.^1^ Maternal antibodies provide protection to a range of infections but they wane over a period of 6-12 months.^2^ Here, we present a case of a 23 days neonate who presented to our centre with dengue fever.

## CASE REPORT

Our patient is a 23 days old male born at term, appropriate for gestational age newborn with uneventful antenatal and postnatal history, delivered vaginally at a maternity hospital. The patient presented to the tertiary care centre with complaints of fever for four days decreased feeding and lethargy with no history of vomiting, fast and noisy breathing, loose stool, and bleeding from any site.

At the day of admission, the baby had a fever of 104°F, heart rate of 130 beats per minute, respiratory rate of 66 breaths per minute, and capillary refill time of less than three seconds with all peripheral pulses palpable. Considering the age, a neonatal sepsis workup was done which showed thrombocytopenia. Renal function test values were within normal limits. Blood culture results were negative. Cerebrospinal fluid analysis was not done because of the low platelets count. After admission, a baby was managed on intravenous cefotaxime and amikacin for five days. Despite the use of antibiotics, the neonate was febrile till the fifth day of admission and platelets count decreased to 30000 platelets per microliter of blood. One pint of PRBC was transfused and the platelet count was increased to 69000 platelets per microliter of blood. The baby not settled to antibiotics was upgraded to injection meropenem. Dengue non-structural protein 1 (NS1) antigen and antibodies IgM were sent on the fourth day of admission, which came positive whereas IgG came negative. Further, the mother was investigated in the lines of dengue suspecting vertical transmission. However, all the tests came negative. Hence, a diagnosis of postnatally acquired dengue was made at this point. The platelets count of the newborn was monitored daily. There was no mucosal bleeding or any internal bleeding. A platelet transfusion was done. Once the fever and the platelet were in normal range, the patient was discharged after 21 days of admission.

## DISCUSSION

Dengue is one of the most frequently encountered viral illnesses and a prevalent public health issue. According to the current epidemiological data, there is a rapid rise in infection rates among children and adults including pregnant women. There are many risks associated with it. In a pregnant woman, it can lead to premature labour.^3^ In addition to that, in cases of vertical transmission resulting in increased perinatal morbidity and mortality.^4^

Dengue is diagnosed by NS1 rapid diagnostic test (RDT). It has 99.2 *%* sensitivity and 96.0% of specificity when analysed using Dengue Virus (DENV) NS1 enzyme-linked immunosorbent assay (ELISA) as standard.^5^ NS1 antigen is detectable in blood from the first day after the onset of fever up to the ninth day. The positive predictive value (PPV) is expected to be greater than 85% in most endemic countries, where dengue accounts for 30% of febrile illnesses.

Infants in the first six months of life are usually protected from dengue infection by maternal antibodies in endemic areas.Although there are several cases of vertical transmission of dengue in the neonatal period reported in the literature.^6-9^ However, responses of postnatally acquired dengue in the neonatal age group are very few typically presenting a clinical picture of very sick bleeding neonates.^10-12^ In our case, the dengue was diagnosed on the fourth day of admission.

Dengue is caused by DENV of the family *Flaviviridae* transmitted by the vector Aedes aegypti mosquito. Dengue has an incubation period of three to eight days but in the newborn period, neonates can become asymptomatic as late as 12 days after birth.^13^ DENV has four serotypes. Following the first episode of infection body produces protective antibodies against the current infecting serotype leaving a possibility of cross-infection. In this case infection by more than one serotype can result in dengue hemorrhagic fever or dengue shock syndrome.^11^ Dengue commonly is a disease in young children. However, the resurgence of dengue in the recent decade is associated with an increased number of infections in the adult population, including pregnant women.

Hence, contributes to the number of neonatal infections due to transplacental transmission during the last trimester of pregnancy. In the case, of vertical transmission, maternal infection leads to viremia and IgM positive in both the mother and neonate.^14^ The risk of DSS/DHF in the neonatal period, though rare can occur when protective antibody titre decrease due to catabolism and cross-reacting and enhancing antibody titre increase.^13^

In the literature, there were reports of neonates developing hemorrhagic manifestations like blood in vomitus and rashes progressing into shock in absence of maternal infection, indicating postnatal transmission.^10-12^

Therefore, keeping the index case in mind, it is suggested that neonatal dengue should be kept as a differential diagnosis for neonatal sepsis and neonatal thrombocytopenia in absence of any evidence of infection in a mother or a woman near-term pregnancy, particularly in endemic areas. The awareness about the possibility of dengue occurring postnatally in the newborn period as a differential diagnosis for neonatal thrombocytopenia and neonatal sepsis is required for the proper management of a healthy neonate.

## Consent:

**JNMA Case Report Consent Form** was signed by the patient and the original article is attached with the patient's chart.
